# Elevated markers of thrombo-inflammatory activation predict outcome in patients with cardiovascular comorbidities and COVID-19 disease: insights from the LEOSS registry

**DOI:** 10.1007/s00392-020-01769-9

**Published:** 2020-11-19

**Authors:** Sebastian Cremer, Carolin Jakob, Alexander Berkowitsch, Stefan Borgmann, Lisa Pilgram, Lukas Tometten, Annika Classen, Kai Wille, Simon Weidlich, Beate Gruener, Stefanie Dimmeler, Steffen Massberg, Siegbert Rieg, Andreas M. Zeiher

**Affiliations:** 1 Department of Medicine III, Cardiology/Angiology/Nephrology, Goethe University of Frankfurt, Theodor-Stern-Kai 7, 60590 Frankfurt, Germany; 2 German Center for Cardiovascular Research DZHK, partner site Rhine-Main, Berlin, Germany; 3 Cardiopulmonary Institute, Goethe University Frankfurt, Frankfurt, Germany; 4 Faculty of Medicine and University Hospital Cologne, Department I for Internal Medicine, University of Cologne, Cologne, Germany; 5 Department of Infectious Diseases and Infection Control, Ingolstadt Hospital, Ingolstadt, Germany; 6 Hospital Ernst Von Bergmann, Potsdam, Germany; 7 Department of Hematology/Oncology, Johannes Wesling Hospital, Minden, Germany; 8 Department of Internal Medicine II, University Hospital Rechts Der Isar, Technical University Munich, Munich, Germany; 9 Department of Infectious Diseases, Internal Medicine III, Ulm, Germany; 10 Institute for Cardiovascular Regeneration, Goethe University Frankfurt, Frankfurt, Germany; 11 Department of Medicine I, Klinikum Der Universität München, Munich, Germany; 12 Department of Internal Medicine, Hematology/Oncology, Goethe University Frankfurt, Frankfurt am Main, Germany; 13 Internal Medicine II, Department of Infectious Diseases, Freiburg University Hospital, Freiburg, Germany

**Keywords:** Inflammation, COVID-19, Myocardial injury, Coagulation, Mortality

## Abstract

**Aims:**

SARS-CoV-2 infection is associated with adverse outcomes in patients with cardiovascular disease. Here, we analyzed whether specific biomarkers predict the clinical course of COVID-19 in patients with cardiovascular comorbidities.

**Methods and results:**

We enrolled 2147 patients with SARS-CoV-2 infection which were included in the Lean European Open Survey on SARS-CoV‑2 (LEOSS)-registry from March to June 2020. Clinical data and laboratory values were collected and compared between patients with and without cardiovascular comorbidities in different clinical stages of the disease. Predictors for mortality were calculated using multivariate regression analysis. We show that patients with cardiovascular comorbidities display significantly higher markers of myocardial injury and thrombo-inflammatory activation already in the uncomplicated phase of COVID-19. In multivariate analysis, elevated levels of troponin [OR 1.54; (95% CI 1.22–1.96), *p* < 0.001)], IL-6 [OR 1.69 (95% CI 1.26–2.27), *p* < 0.013)], and CRP [OR 1.32; (95% CI 1.1–1.58), *p* < 0.003)] were predictors of mortality in patients with COVID-19.

**Conclusion:**

Patients with cardiovascular comorbidities show elevated markers of thrombo-inflammatory activation and myocardial injury, which predict mortality, already in the uncomplicated phase of COVID-19. Starting targeted anti-inflammatory therapy and aggressive anticoagulation already in the uncomplicated phase of the disease might improve outcomes after SARS-CoV-2 infection in patients with cardiovascular comorbidities.

**Graphic abstract:**

Elevated markers of thrombo-inflammatory activation predict outcome in patients with cardiovascular comorbidities and COVID-19 disease: insights from the LEOSS registry

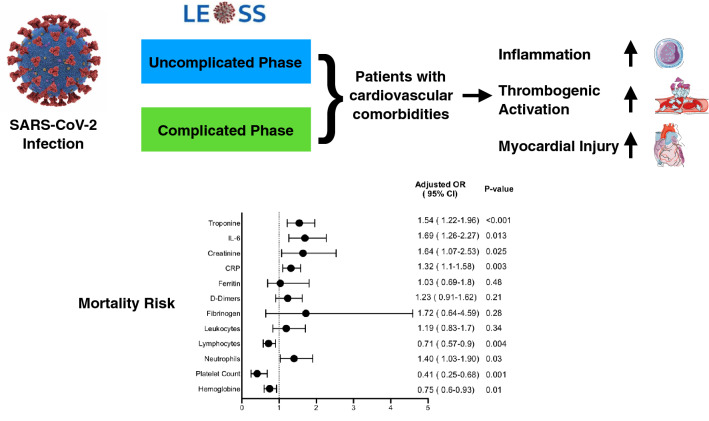

**Electronic supplementary material:**

The online version of this article (10.1007/s00392-020-01769-9) contains supplementary material, which is available to authorized users.

## Introduction

Infection with the novel coronavirus SARS-CoV-2 causes the so-called coronavirus disease COVID-19, which has become a worldwide pandemic [1]. Based on initial reports from the outbreak of the disease in China, COVID-19 progression is characterized by three distinct phases: an initial infection phase followed by a respiratory distress phase and finally culminating in a severe hyperinflammatory state [2]. While more than 80% of SARS-CoV-2 infections show only mild or even absent symptoms, the cardiovascular system has been documented to play an important role both as a primary target as well as a risk amplifying comorbidity factor for COVID-19 [3–6]. In fact, a very recent study with more than 2700 laboratory confirmed COVID-19 patients revealed that patients with preexisting cardiovascular disease (CVD) are more likely to experience myocardial injury as measured by increased troponin release, which by itself is associated with a profound increase in risk for mortality from COVID-19 [7]. However, major gaps remain to understand the pathophysiological mechanisms, by which infection with SARS-CoV-2 leads to cardiovascular morbidity and associates with worse clinical outcome in patients with preexisting cardiovascular disease.

Therefore, it was the aim of the present study to decipher specific properties of patients with cardiovascular disease suffering from COVID-19 and to address whether these characteristics contribute to worse clinical outcome during COVID-19 progression. Specifically, we were interested in finding out if cardiac and inflammatory biomarkers in patients with COVID-19 would predict disease outcomes. To do so, we used data from one of the largest European COVID-19 registries, the Lean European Open Survey on SARS-CoV-2 (LEOSS) registry (www.leoss.net) [8].

## Methods

### Study population

**Fig. 1:**
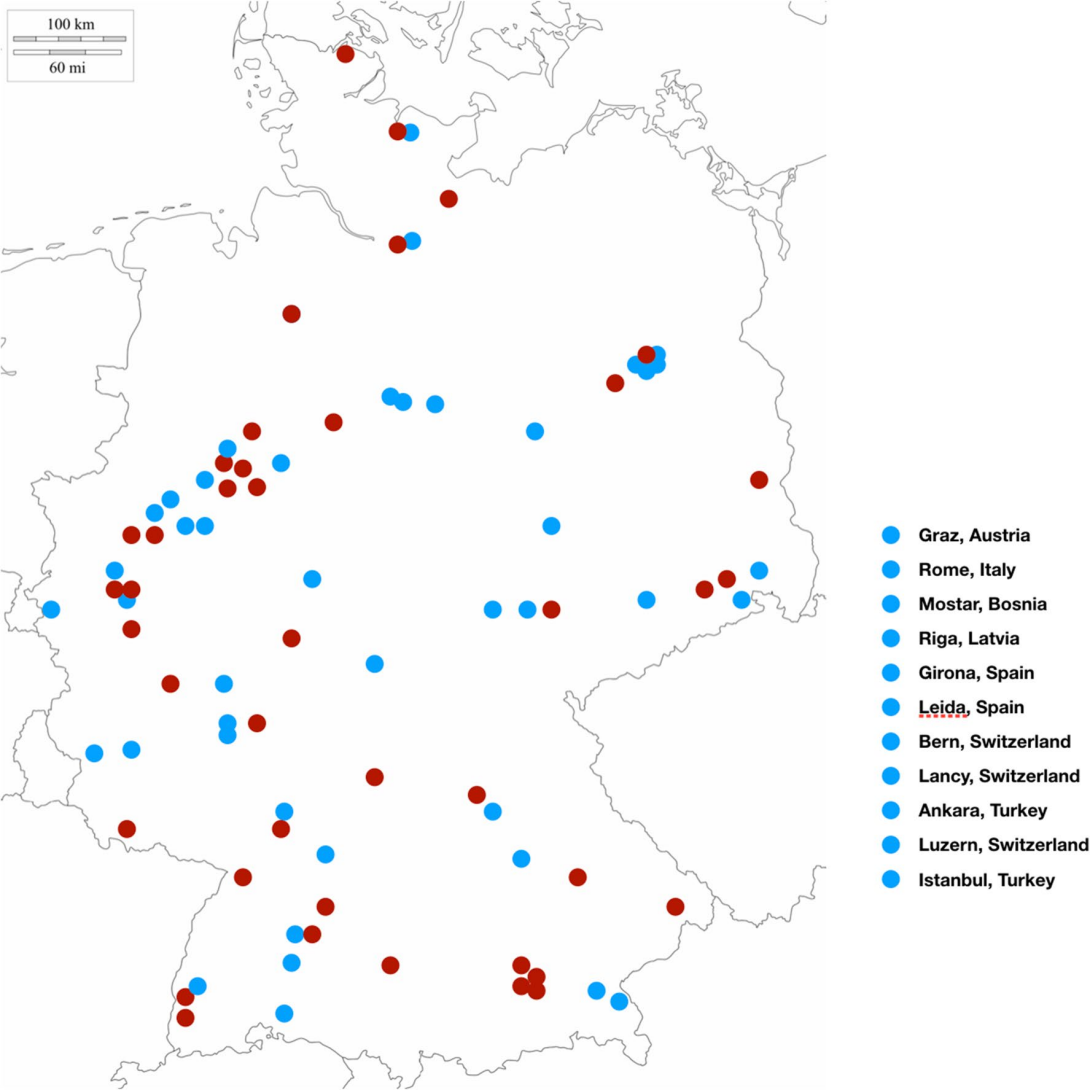
Map with all centers which added patients to the study cohort. Centers which included patients with cardiovascular comorbidities are highlighted in red

The study population consists of 2147 consecutive patients, who were included in the LEOSS (Lean European Open Survey on SARS-CoV-2) registry between 03/18/2020 and 06/08/2020 across 122 hospitals in Europe, most of them in Germany. A map with all recruiting centers is shown in Fig. 1. All patients had a diagnosis confirmed by positive results of PCR testing. Data collection was performed retrospectively and anonymously, while only data from standard of care treatment are documented. Patients were stratified into the following groups at an initial positive test result for SARS-CoV-2: patients in the *uncomplicated phase* were either asymptomatic, and had symptoms of upper respiratory tract infection, fever or nausea, emesis, or diarrhea. Patients in the *complicated phase* had at least one of the following characteristics: new need for oxygen supplementation or clinically relevant increase of prior oxygen home therapy, PaO2 at room air < 70 mmHg, SO2 at room air < 90%, increase of AST or ALT > 5 × ULN (upper limit of normal), new cardiac arrythmia, new pericardial effusion > 1 cm or new heart failure with pulmonary edema, congestive hepatopathy, or peripheral edema. Patients in the *critical phase* were dependent on catecholamines, experienced life-threatening cardiac arrhythmia, had mechanical ventilation (invasive or non-invasive), or need for unplanned mechanical ventilation prolongation (> 24 h) of planned mechanical ventilation, liver failure with an INR > 3.5 (quick < 50%), a qSOFA score of >  = 2, or acute renal failure with need of dialysis. This study was approved by the responsible ethics committee of all participating study sites.

### Data collection

Demographic, clinical, laboratory, treatment, and outcome data were extracted from the in-hospital medical records. Analyzed laboratory data were collected within 48 h of a positive PCR result irrespective of the patient’s status. Therefore, all biomarker measurements represent baseline values. Data were distributed into different categories. Cardiovascular (CV) comorbidity was defined by one of the following diseases: coronary artery disease, prior myocardial infarction, chronic heart failure, atrial fibrillation, AV-Block, and aortic valve stenosis. As all data are based on anonymous reports, patients can be included without informed consent. Several steps are taken to prevent re-identification which include vertical (categorical assessment of numerical variables) and horizontal data aggregation (data aggregation within the phases of disease). The median of documented cases per center is seven in the entire cohort (range 1–181) and eight (range 3–60) in the subset of patients with cardiovascular comorbidities.

### Statistical analysis

All data are presented as categorical variables (values and proportions) and were analyzed with Chi-square or Fisher’s exact test, where appropriate. Outcome in patients being in the non-critical phase at baseline was analyzed with univariate binary regression analysis and parameters associated with outcome were further included in multivariate analysis for identification of independent predictors. The multivariate analysis was performed using logistic regression according to Wald approach in step-down mode. Primary endpoint of the study was death of any cause. Death or reaching a critical condition in patients being not critical when testing positive for SARS-CoV-2 was considered as combined endpoint. For all the statistical analyses, *p* < 0.05 was considered to be significant. Analyses were performed with SPSS, version 26 (IBM, Chicago, Illinois).

## Results

### Characteristics of patients at baseline

First, patients were stratified according to disease stage after being tested positive for SARS-CoV-2. Among a total of 2147 patients, 1,343 (62.55%) were in the uncomplicated phase of COVID-19, 641 (29.86%) in the complicated phase and 163 (7.59%) patients in the critical phase. Increased disease severity correlated with age (*p* < 0.001), which was associated with male gender (*p* = 0.021) and a higher BMI (*p* < 0.001). Of note, patients who presented in a complicated or critical phase of the disease were more likely to suffer from cardiovascular comorbidities (*p* < 0.001), including coronary artery disease (*p* = 0.011), chronic heart failure (*p* = 0.001), and atrial fibrillation (*p* = 0.001). In addition, they were more likely to have hypertension, diabetes mellitus, chronic kidney disease (*p* < 0.001 for all), or a history of cancer (*p* = 0.043), and were more often smokers (*p* = 0.032; Table 1).

**Fig. 2:**
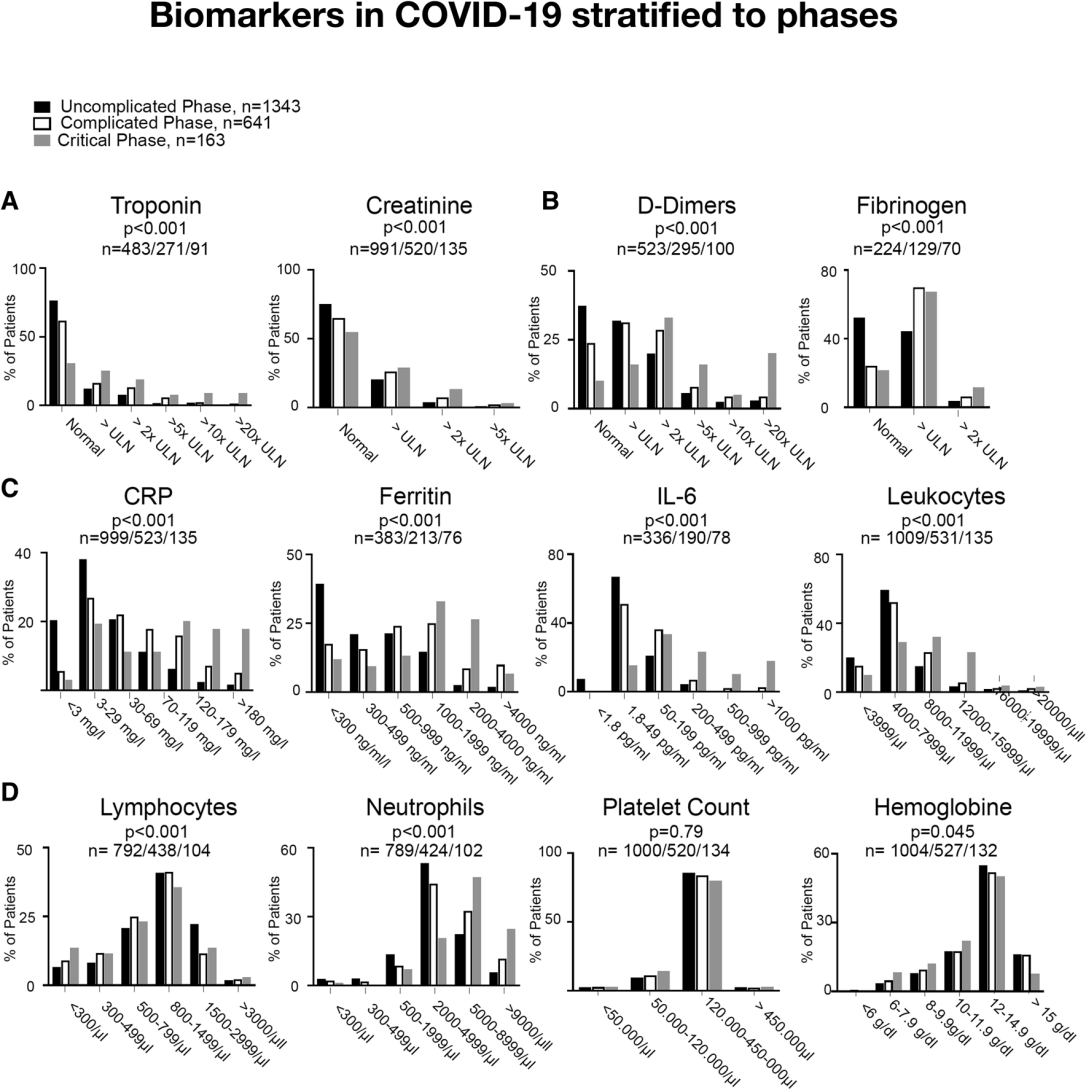
Serum levels of Troponin and Creatinine (**a**), d-dimers and Fibrinogen (**b**), CRP, IL-6, Ferritin, and WBC (**c**), Lymphocyte and Neutrophil counts, platelet count, and hemoglobin (**d**) of patients in the whole-study cohort who presented in the uncomplicated phase (black bars), the complicated phase (white bars), and the critical phase (gray bars). n-numbers of available lab values in each group and *p* values are depicted in each panel

Next, we compared inflammatory and cardiovascular biomarkers within patients who presented in the uncomplicated, the complicated, and the critical phase, respectively. Here, we report higher levels of IL-6, CRP, ferritin, leukocytes, and neutrophils in patients with progressed COVID-19 disease (*p* < 0.001 for all). Lymphocyte levels (*p* < 0.001) and hemoglobin values (*p* = 0.045) were lower in more severe cases. Interestingly, also markers of cardiac damage and thrombogenic activation were elevated in cases with severe COVID-19 infection. Specifically, we report higher levels for troponin, fibrinogen, and d-dimers (*p* < 0.001 for all, Fig. 2).

### Biomarkers in patients with cardiovascular disease and COVID-19 according to disease phase

**Fig. 3:**
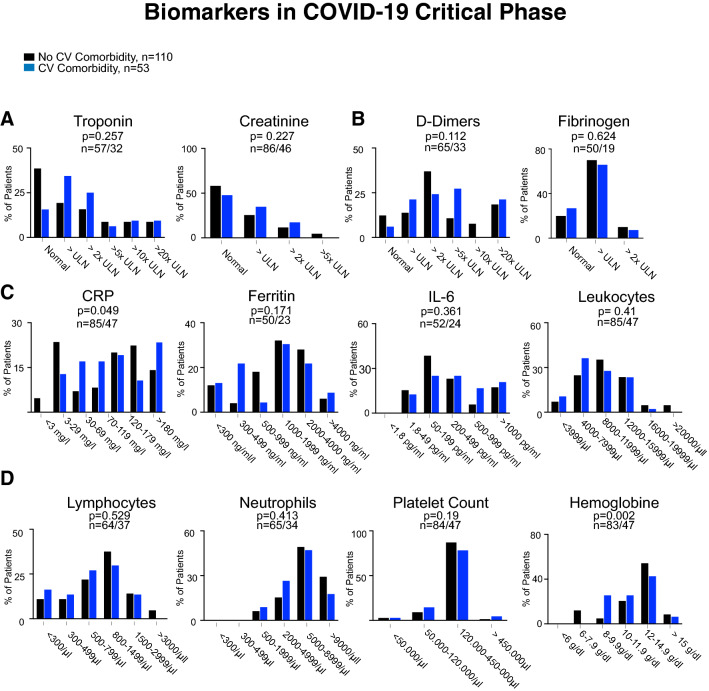
Serum levels of Troponin and Creatinine (**a**), d-dimers and Fibrinogen (**b**), CRP, IL-6, Ferritin, and WBC (**c**), and Lymphocyte and neutrophil counts, platelet count, and hemoglobin (**d**) in patients who presented in the critical phase with no cardiovascular comorbidity (black bars) and cardiovascular comorbidity (blue bars). n-numbers in patients without cardiovascular comorbidities compared to patients with cardiovascular disease and p values are depicted in each panel

**Fig. 4:**
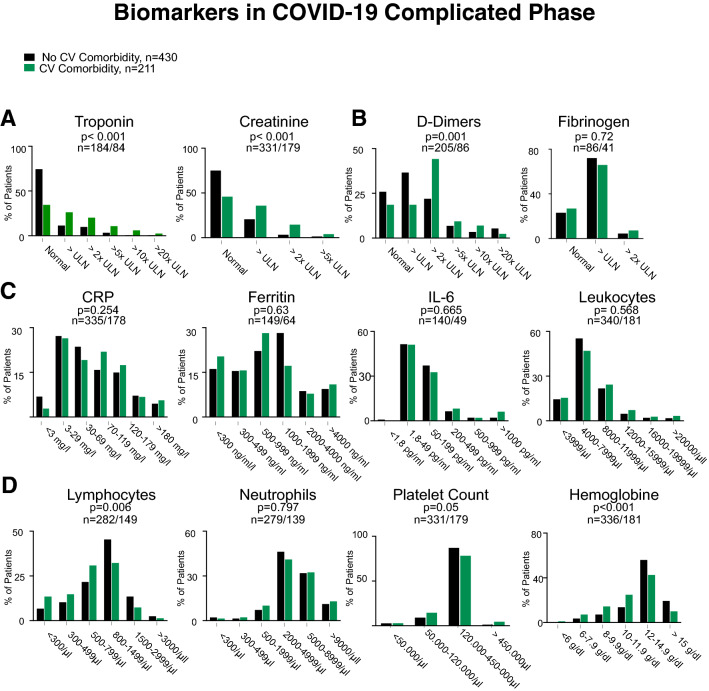
Serum levels of Troponin and Creatinine (**a**), d-dimers and Fibrinogen (**b**), CRP, IL-6, Ferritin and WBC (**c**), Lymphocyte and neutrophil counts, platelet count, and hemoglobin (**d**) in patients who presented in the complicated phase with no cardiovascular comorbidity (black bars) and cardiovascular comorbidity (green bars). n-numbers in patients without cardiovascular comorbidities compared to patients with cardiovascular disease and *p* values are depicted in each panel

**Fig. 5:**
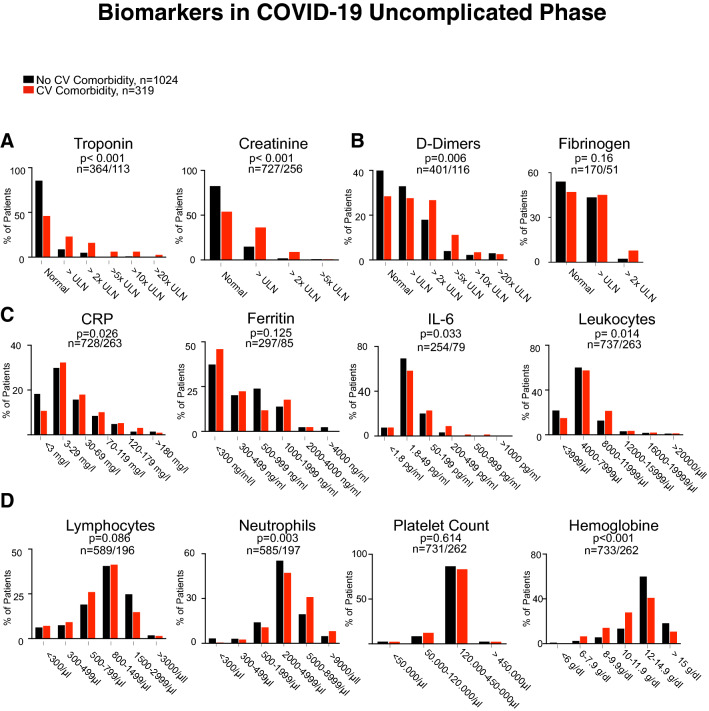
Serum levels of Troponin and Creatinine (**a**), d-dimers and Fibrinogen (**b**), CRP, IL-6, Ferritin, and WBC (**c**), and Lymphocyte and Neutrophil counts, platelet count, and hemoglobin (**d**) in patients who presented in the uncomplicated phase with no cardiovascular comorbidity (black bars) and cardiovascular comorbidity (red bars n-numbers in patients without cardiovascular comorbidities compared to patients with cardiovascular disease and p values are depicted in each panel

Hyperinflammation is a hallmark of severe COVID-19 disease, in which myocardial injury occurs frequently and predicts death. Patients with preexisting cardiovascular disease have worse outcomes in COVID-19 [7]. Therefore, we compared biomarkers in the different phases of the disease between patients with cardiovascular comorbidities and patients without an underlying cardiovascular condition. Here, we did not detect significant differences in biomarker levels in the critical phase, except higher CRP values in patients with CVD (Fig. 3). However, in the complicated phase, markers of myocardial injury (troponin, *p* < 0.001), kidney damage (creatinine, *p* < 0.001), and thrombogenic activation (d-dimers, *p* = 0.001) were elevated in patients with CVD, whereas markers of inflammation did not differ (Fig. 4). In the uncomplicated phase, when patients were either asymptomatic or had mild respiratory symptoms, this was very different. Here, patients with cardiovascular comorbidities were more likely to have elevated levels of IL-6 (*p* = 0.033), CRP (*p* = 0.026), and higher leukocyte counts (p = 0.014) in addition to markers of myocardial injury (Fig. 5). This indicates that patients with underlying cardiovascular disease are more likely to display a systemic inflammatory state already in the uncomplicated phase of the disease. In addition, d-dimers were significantly increased in these patients indicating activation of intravascular coagulation (Fig. 5).

### Outcomes and predictors

Of the 2147 patients included in this analysis, 355 (16.53%) died during follow-up. 9.08% (122) of patients who presented in the uncomplicated phase of the disease died, whereas 24.8% (159) and 45.4% (74) of patients who were initially in the complicated and critical phase at baseline, respectively, died.

**Fig. 6:**
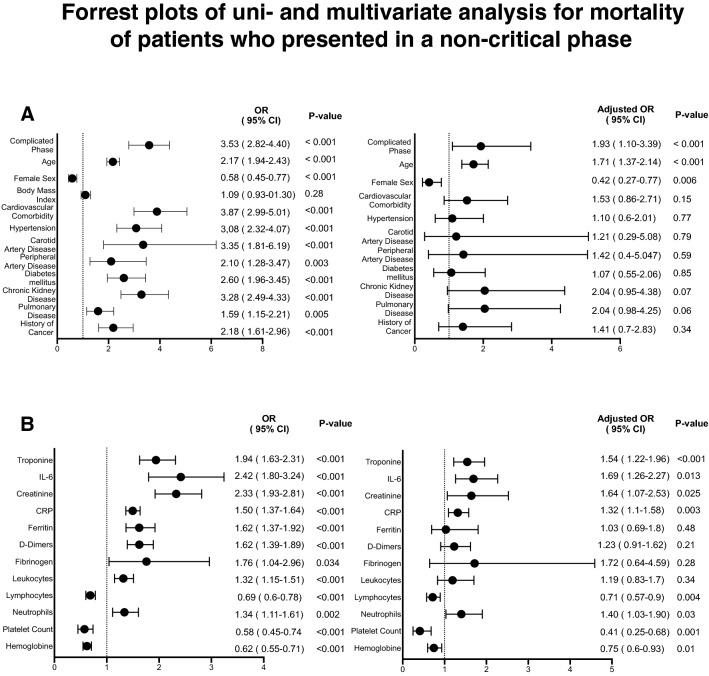
Forrest plots of univariate and multivariate analysis of clinical indicators (**a**) and biomarker alterations (**b**) for mortality in patients who were initially in a non-critical phase considering disease severity (uncomplicated or complicated) at baseline

To analyze the risk factors for mortality in patients, who presented in a non-critical phase of COVID-19, univariate binary regression analysis was performed. Here, higher disease severity, sex and age, cardiovascular comorbidity, hypertension, carotid and peripheral artery disease, as well as diabetes mellitus, chronic kidney disease, pulmonary disease, and a history of cancer were associated with an increased risk of death. In this analysis, all biomarkers evaluated qualified to assess mortality risk. To define independent predictors of death in patients with COVID-19, multivariate analysis including clinical severity stage was conducted. Here, only a complicated phase of COVID-19 at baseline [OR 1.93 (95% CI 1.10–3.39); *p* < 0.001] and the demographic variables sex [OR 0.42 (95% CI 0.27–0.77); *p* = 0.006] and age [OR 1.71 (95% CI 1.37–2.14); *p* < 0.001] were independent predictors of mortality, whereas all comorbidities, including cardiovascular disease, lost their predictive power (Fig. 6a).

Among biomarkers, elevated troponin levels were the strongest predictor of death [OR 1.54; (95% CI 1.22–1.96); *p* < 0.001], followed by IL6-levels [OR 1.69 (95% CI 1.26–2.27); *p* < 0.013] and CRP [OR 1,32; (95% CI 1.1–1.58); *p* < 0.003]. In addition, creatinine and higher total leukocyte count and neutrophil levels as well as lower levels of hemoglobin and platelets where independent predictors of mortality in multivariate analysis. Here, all other markers lost their predictive significance (Fig. 6b).

## Discussion

The results of the present analysis from a very large European Registry on COVID-19, the LEOSS registry, for the first time demonstrate that patients with cardiovascular comorbidities exhibit increased thrombo-inflammatory serum markers already during the uncomplicated phase of COVID-19, suggesting an enhanced susceptibility for thrombo-inflammatory activation following SARS-CoV-2 infection. In addition to confirming the previously extensively described prognostic importance of myocardial injury (as assessed by troponin release) for overall mortality, our data provide further support for a hyperinflammatory response induced by SARS-CoV-2 as a major determinant of disease severity and death.

Pathological inflammation plays a key role in COVID-19 [9]. Specifically, Nicolai et al. elegantly demonstrated a key role for immunothrombotic dysregulation for disease severity and multi-organ involvement due to inflammatory microvascular thrombi in COVID-19 [10]. In accordance with previous reports, the results of the present study document that thrombo-inflammatory markers in blood were associated with disease progression and mortality [4, 11, 12]. Most importantly, however, patients with cardiovascular comorbidities defined as coronary artery disease, chronic heart failure, and atrial fibrillation displayed an elevated thrombo-inflammatory state as evidenced by significantly elevated levels of d-dimers, CRP, IL-6, and neutrophils already at admission in the uncomplicated phase of COVID-19. On multivariate analysis in addition to troponin, IL-6 and CRP serum levels remained significant predictors of mortality in patients initially admitted to the hospital in a non-critical phase, whereas the presence of cardiovascular comorbidity was no longer independently associated with an adverse clinical course.

Thus, it appears that the enhanced susceptibility for thrombo-inflammatory activation is the major driver for worse outcome in patients with cardiovascular comorbidities following infection with SARS-CoV-2. Mechanistically, the proinflammatory cytokine IL-6 exerts a central function to initiate inflammation induced coagulation via the expression of tissue factor on both mononuclear cells as well as the vascular endothelium [9], contributing to the formation of microthrombi, a hallmark of organ injury not only in sepsis, but also increasingly described in COVID-19 [13–15]. Activated inflammatory cells are increasingly recognized to contribute to cardiovascular disease [16]. Atherosclerosis, heart failure, and atrial fibrillation are well established to be associated with elevated inflammation markers including IL-6 [17, 18]. Moreover, the recent discovery of somatic mutations in hematopoietic cells (“clonal hematopoiesis of indeterminate potential (CHIP)”), which is associated with increased aggressiveness of inflammatory cells and is significantly enriched in patients with cardiovascular disease, does provide a further link connecting aging and inflammation in cardiovascular disease [19]. Indeed, our recent single-cell RNA-sequencing study revealed that monocytes of patients with cardiovascular disease carrying CHIP driver mutations are primed for exuberant inflammatory responses and IL-6 production [20]. Taken together, patients with cardiovascular disease are sensitized for inflammatory activation, which may contribute to worse clinical outcomes following infection with SARS-CoV-2.

Importantly, IL-6 is not only an important biomarker and possible target for cardiovascular morbidity and mortality, but may also serve as a therapeutic target in patients with COVID-19 [21]. Indeed, clinical trials assessing the benefits of inflammatory cytokine blockade targeting IL-6 and its upstream mediator IL-1ß are in progress [9]. Given the heterogeneous preliminary results so far in trials applying IL-6 blockade in more severe cases of COVID-19 [22, 23], the results of the present study may suggest to elucidate the specific role of IL-6 blockade in uncomplicated COVID-19 patients with cardiovascular comorbidities. Likewise, in light of the observed enhanced intravascular coagulation even during the uncomplicated phase of COVID-19 in patients with cardiovascular comorbidities, more aggressive anticoagulation treatment strategies may be warranted in these patients, as suggested in a recent retrospective analysis [24].

## Limitations

By virtue of the design of the LEOSS registry to anonymously capture patient data, we cannot provide absolute values for the individual biomarkers, which were divided into different categories. In addition, we do not have sequential biomarker measurements. Thus, we cannot comment on the possible role of dynamic alterations of individual biomarkers in the process of disease progression. However, we believe that it is important to risk stratify COVID-19 patients at the time of initial presentation to facilitate the induction of specific treatment strategies. Finally, cause of death was not analyzed in the present cohort.

In summary, patients with cardiovascular comorbidities are characterized by an enhanced thrombo-inflammatory activation already during the uncomplicated phase of COVID-19, which associates with worse outcome and increased mortality. Future studies should address a potential benefit of targeted anti-inflammatory therapy and aggressive anticoagulation in patients with cardiovascular comorbidities infected with SARS-CoV-2 already during the uncomplicated phase of COVID-19.

**Table 1:** Baseline characteristics of patients in an uncomplicated, complicated or critical phase of COVID-19 disease at day of an initial positive test result for SARS-CoV-2

	Total	Uncomplicated phase	Complicated phase		Critical phase	*P* value
	*N* = 2147	*N *= 1343 (62.5%)	*N* = 641 (29.86%)		*N *= 163 (7.59%)	
Demographics						
Age, years						
< 25 years	67 (3.12%)	61 (4.18%)	3 (0.47%)		3 (1.84%)	<0.001
26–35 years	133 (6.19%)	110 (8.19%)	20 (3.12%)		3 (1.84%)	
36–45 years	172 (8.01%)	126 (9.38%)	38 (5.93%)		8 (4.91%)	
46–55 years	326 (15.18%)	206 (15.34%)	93 (14.51%)		27 (16.56%)	
56–65 years	412 (19.19%)	266 (19.81%)	110 (17.16%)	36 (22.09%)		
66–75 years	370 (17.23%)	204 (15.19%)	127 (19.81%)	39 (23.93%)		
76–85 years	491 (22.87%)	277 (20.63%)	177 (27.61%)	37 (22.70%)		
> 85 years	176 (8.20%)	93 (6.92%)	73 (11.39%)		10 (6.13%%)	
Sex (male), *n* (%)	1286 (59.90%)	783 (58.30%)	390 ( 60.84%)		113 (69.33%)	0.021
Body mass index (kg/m^2^); *n*=1258						
< 18.5	37 (2.94%)	26 (1.94%)	6 (0.94%)		5 (3.07%)	<0.001
18.5–24.9	470 (37.36%)	299 (22.26%)	145 (22.62%)	26 (15.95%)		
25–29.9	449 (35.69%)	260 (19.36%)	156 (24.34%)	33 (20.25%)		
30–34.9	219 (10.20%)	122 (9.08%)	77 (12.01%)		20 (12.27%)	
>35	106 (8.42%)	46 (3.43%)	39 ( 6.08%)		21 (12.88%)	
Clinical history						
Cardiovascular comorbidities	583 (27.15%)	319 (23.75%)	211(32.92%)		53 (32.52%)	< 0.001
Coronary artery disease	303 (14.11%)	168 (12.51%)	108 (16.85%)		27 (16.56%)	0.011
Chronic heart failure	216 (10.06%)	112 (8.34%)	83 (12.95%)		21(12.88%)	0.001
Atrial fibrillation	291 (13.55%)	155 (11.54%)	113 (17.63%)		23 (14.11%)	0.001
AV-Block	53 (2.47%)	32 (2.38%)	18 (2.81%)		3 (1.84%)	0.696
Aortic valve stenosis	56 (2.61%)	31 (2.31%)	23 (3.59%)		2 (1.23%)	0.112
Hypertension	1043 (48.58%)	578 (43.04%)	363 (56.63%)		102 (62.58%)	< 0.001
Peripheral artery disease	95 (4.42%)	50 (3.72%)	41 (6.4%)		4 (2.45%)	0.008
Carotid artery disease	49 (2.28%)	19 (1.41%)	29 (4.52%)		1 (0.61%)	< 0.001
Diabetes	408 (19.0%)	213 (15.86%)	146 (22.78%)		49 (30.06%)	< 0.001
Chronic kidney disease	382 (17.79%)	201 (14.97%)	153 ( 23.87%)		28 (17.18%)	< 0.001
Chronic pulmonary disease	314 (14.62%)	171 (12.73%)	111 (17.32%)		32 (19.63%)	0.006
Cancer	269 (14.56%)	191 (14.22%)	109 ( 17.0%)		16 (9.82%)	0.043

## Electronic supplementary material

Below is the link to the electronic supplementary material.

Supplementary file1 (DOCX 158 KB)
