# Supplementary material for: Elevated markers of thrombo-inflammatory activation predict outcome in patients with cardiovascular comorbidities and COVID-19 disease: insights from the LEOSS registry
Source: Clin Res Cardiol. 2020 Nov 19;110(7):1029–40. doi: 10.1007/s00392-020-01769-9 (PMC7674577; doi:10.1007/s00392-020-01769-9)
Supplement: Supplementary file 1 — Supplementary file1 (DOCX 158 KB) [file 392_2020_1769_MOESM1_ESM.docx]

To

Prof. Dr. Michael Böhm

*Clinical Research in Cardiology*

Re: Submission of an original investigation to *Clinical Research in Cardiology*

Dear Prof. Böhm, dear Michael,

enclosed we would like to submit our manuscript entitled

*“***Elevated markers of thrombo-inflammatory activation predict outcome in patients with cardiovascular comorbidities and COVID-19 disease: insights from the LEOSS registry***”*

for publication in *Clinical Research in Cardiology* as an original manuscript*.*

Infection with the novel corona virus SARS-CoV-2 causes the so-called coronavirus disease COVID-19, which has become a worldwide pandemic. Deciphering mechanisms of disease progression, including identifying specific populations who display increased risk of experiencing a complicated course of the disease is of paramount importance for society and medicine. The cardiovascular system has been documented to play an important role both as a primary target as well as a risk amplifying comorbidity factor for COVID-19. However, mechanisms which contribute to this risk amplification are unclear.

In the present manuscript we demonstrate in one of the largest cohorts of patients with SARS-CoV-2 infection, the LEOSS registry, that

1) patients with cardiovascular comorbidities display significantly higher markers of myocardial injury and thrombo-inflammatory activation already in an uncomplicated phase of COVID-19
2) elevated levels of troponin are the strongest predictor of mortality in patients with COVID-19.

We believe that these findings are of broad interest to the readership of your journal, as it is relevant to cardiovascular clinicians and to the immunology, hematology and basic science community. All authors have agreed to the submission of the manuscript, and there is no conflict of interest.

Therefore, we do believe that Clinical Research in Cardiology is ideally suited for such a report.

Looking forward to a positive response.

Sincerely,

Andreas M. Zeiher, MD

Professor of Medicine

Goethe University

Frankfurt

Germany
